# Surface-Mediated Modulation of Different Biological Responses on Anatase-Coated Titanium

**DOI:** 10.3390/jfb15020029

**Published:** 2024-01-25

**Authors:** Leila Mohammadnejad, Antonia Theurer, Julia Alber, Barbara Illing, Evi Kimmerle-Mueller, Jacob Schultheiss, Stefanie Krajewski, Frank Rupp

**Affiliations:** Department Medical Materials Science & Technology, Institute for Biomedical Engineering, University Hospital Tübingen, Osianderstr. 2-8, 72076 Tübingen, Germany

**Keywords:** anatase, dental implant, osseointegration, fibroblast

## Abstract

Various surface modification strategies are being developed to endow dental titanium implant surfaces with micro- and nano-structures to improve their biocompatibility, and first of all their osseointegration. These modifications have the potential to address clinical concerns by stimulating different biological processes. This study aims to evaluate the biological responses of ananatase-modified blasted/etched titanium (SLA-anatase) surfaces compared to blasted/acid etched (SLA) and machined titanium surfaces. Using unipolar pulsed direct current (DC) sputtering, a nanocrystalline anatase layer was fabricated. In vitro experiments have shown that SLA-anatase discs can effectively promote osteoblast adhesion and proliferation, which are regarded as important features of a successful dental implant with bone contact. Furthermore, anatase surface modification has been shown to partially enhance osteoblast mineralization in vitro, while not significantly affecting bacterial colonization. Consequently, the recently created anatase coating holds significant potential as a promising candidate for future advancements in dental implant surface modification for improving the initial stages of osseointegration.

## 1. Introduction

Millions of people around the world suffer from edentulism, a condition characterized by the loss of one or more teeth up to complete edentulous maxillae or mandibulae [1]. Over the last 30 years, the number of dental implants has risen to more than five million per year, making them the treatment of choice for oral rehabilitation [2,3,4]. Titanium implants were introduced in the 1960s and have now become the most common type of dental implant [5,6]. Originally, titanium implants were made of commercially pure titanium (cpTi) that was anchored within bone tissue by direct contact, which is also known as “osseointegration” [7,8,9]. Currently, most dental implants are made from titanium and its alloys, which are highly successful and rarely cause complications [10]. The high success rate of titanium-based implants is attributed to a variety of factors, including the reliability of their mechanical properties, bone-contact biocompatibility, and their ability to osseointegrate [11,12,13]. Although dental implants have a high survival and success rate [6,14,15,16], early implant failure due to poor osseointegration occurs in 1–4% of cases [4,16,17,18]. Also, infection and inflammation in the peri-implant region (peri-implantitis) can result in implant failure [19].

In terms of the biocompatibility and osseointegration of titanium implants, surface properties have been found to have a significant impact. Surface modifications are therefore the most common strategy used to improve the titanium implant’s osseointegration capacity [8,18]. A variety of surface modification approaches have been developed over the past decades to promote biocompatibility and osseointegration, as well as to accelerate the healing process and prevent the formation of bacterial biofilms [8,20,21,22]. Different additive and subtractive methods have been used to modify surfaces, such as grit blasting, acid etching, and anodizing [8]. The titanium metallic bulk is naturally covered by a titanium oxide layer (TiO_2_), which contributes to its biocompatibility and osseointegration features. Furthermore, several studies have shown that the composition, morphology, and structure of this titanium dioxide passivation layer determine titanium implants’ chemical resistance [17,23]. In addition to its amorphous state, titanium dioxide has three different crystal lattices, anatase, rutile, and brookite, two of which are distributed naturally on the titanium surface (anatase and rutile) [23,24,25]. Among the three conformations, anatase is the most stable form, and produces nanocrystalline titanium dioxide at a relatively low temperature [25]. Several methods allow the preparation and deposition of anatase thin films on different substrates, such as chemical vapor deposition (CVD) techniques, physical vapor deposition (PVD), as well as wet chemical deposition techniques [26,27]. PVD is a thin-film atomic deposition process performed in a vacuum, gaseous, plasma, or electrolytic environment [28]. PVD processes in gaseous states include evaporation and sputtering, the latter allowing for the better control of film thickness [28]. Our group investigated the physico-chemical properties of nanocrystalline anatase thin films prepared by reactive pulse magnetron sputtering [29], and showed in further studies an increased formation of macromolecular salivary pellicles [30] as well as improved osteoblast responses compared to uncoated titanium reference surfaces [17].

Further studies have shown that surfaces rich in anatase increase osteoblast activity [31,32,33], enhance osseointegration [34,35], and promote the deposition of apatite [24,31,33]. A titanium surface coated with anatase also reduces bacterial adhesion and consequent biofilm formation [36,37,38,39]. Different approaches have been developed to coat titanium surfaces with anatase, resulting in promising in vitro and in vivo results [13,17,35].

Based on the above study results regarding anatase surfaces at biological interfaces, the aim of this study is to thoroughly investigate different in vitro biological responses of anatase, including hemocompatibility, cytotoxicity, osteoblast responses, and bacterial interactions. We hypothesize that anatase thin films could considerably improve dental implants’ main features and offer potential clinical value.

## 2. Materials and Methods

### 2.1. Samples and Surface Preparation

Machined, round c.p. titanium grade 4 discs, 10 mm in diameter and 1 mm thick, were included in this study and served as the control group (machined). The machined discs were modified by grit-blasting and acid-etching, resulting in a microrough sample group (SLA). In detail, the materials were first blasted with a medical-grade corundum and then subjected to a thermo-chemical process in a strong acid bath, resulting in a microroughened surface with an average roughness of Sa (arithmetical mean height) of 1–3 µm. This group resembles the blasted-etched surface from commercial SLA implants (medentis medical, Bad-Neuenahr-Ahrweiler, Germany), which is termed SLA in this study.

The microrough discs were further modified in a unipolar pulsed DC sputtering process. Dental implants with this new coating are equipped in the anchoring area with a photoactivatable, nanocrystalline anatase layer of approximately 250–500 nm thickness. First, the SLA discs were pre-treated with argon plasma, which includes surface cleaning and plasma polishing. The titanium dioxide layer is then applied using a unipolar pulsed, reactive (intake of oxygen) magnetron sputtering process (reactive pulse magnetron sputtering, PMS). The sputtering process is followed by the thermal annealing post-treatment of the substrates according to a proprietary process of medentis medical GmbH, Bad-Neuenahr-Ahrweiler, Germany. The respective experimental discs modified by this anatase coating are referred to as SLA-anatase in this study. All samples were prepared and provided by medentis medical, Bad-Neuenahr-Ahrweiler, Germany. Prior to further analyses, all samples were ultrasonically cleaned for 5 min in 70% ethanol and autoclaved.

### 2.2. Physico-Chemical Characterization

#### 2.2.1. Wettability Analysis

The hydrophilicity of the samples was assessed by measuring the static water contact angles using a high-resolution drop shape analysis system (OCA 200, Dataphysics, Filderstadt, Germany). Accordingly, 1 μL drops of ultrapure water (Milli-Q; Merck Millipore, Darmstadt, Germany) were deposited on the sample surface, and after 10 s, the contour of the drop was analyzed using the measurement software (SCA202 V. 6.1.11, Dataphysics, Filderstadt, Germany) and the corresponding contact angle was calculated by applying ellipse fitting to the contour line. A total of five disks and three drops per disc were analyzed for each surface.

#### 2.2.2. Surface Roughness

The roughness of all surface variants was measured using optical 3D confocal microscopy (MarSurf CM Explorer, Mahr, Göttingen, Germany). The measurement of each test disc was conducted in 6 measurement squares of 0.64 mm^2^ each (0.8 mm edge length) using a 20× objective. Roughness was analyzed using MountainsMap Imaging Topography Software (Version 9.1.9957, Digital Surf, Besançon, France). First, the surface was leveled using the least square plane leveling method, and then an S-Filter (λs) (Gauss 300:1 (800 µm)) was applied to remove short-scale components introduced by instrument and environmental noise and an L-Filter (λc) (Gauss (0.05 mm)) was applied to minimize possible waviness. Sa, Sz (maximum heights) and Sdr (the developed interfacial area ratio) were calculated from measurements taken on 5 sample disks per surface variant.

#### 2.2.3. Characterization of Surface Structure

The topography of the implant screws was also examined using a field emission scanning electron microscope (FE-REM, JSM-6500F, Jeol, Tokyo, Japan). Accordingly, implant screws were directly measured at their heads above the threads (measuring point a), in their middles between the threads (measuring point b), and at their tips at the gouges (measuring point c). As a result, two images were taken of each implant at magnifications of 100×, 1000×, 3000×, 5000×, and 10,000×.

### 2.3. Cytotoxicity Testing with L929 Fibroblasts

The extract cytotoxicity test was performed using mouse fibroblast cells (L929, DSMZ GmbH, Braunschweig, Germany) according to ISO 10993-5: 2009 [40]. Accordingly, L929 fibroblasts were cultured in Dulbecco’s modified eagle medium (DMEM, Life Technologies, Paisley, UK) supplemented with 10% heat-inactivated fetal bovine serum (FBS, Life Technologies, Paisley, UK), 1% penicillin/streptomycin (P/S, Life Technologies, Grand Island, NY, USA) and 1% GlutaMAX (Life Technologies, Paisley, UK) under standard conditions (37 °C, 5% CO_2_). Sample extracts were prepared in accordance with ISO 10993-12: 2012 [40]. Consequently, discs were immersed in cell culture medium with FBS (ratio of surface area to extraction medium: 3.0 cm^2^/mL) under cell culture conditions and agitated for overnight. Extracts of the Ti disk and pure Cu extract were used as positive and negative controls, respectively. L929 cells were seeded at a density of 3 × 10^4^ cells/cm^2^ in 96-well plates and incubated overnight at 37 °C. Afterwards, the obtained extracts were added to the culture medium of the L929 cells in different concentrations: high (h)—75% by volume; medium (m)—25% by volume; low (l)—7.5% by volume. Afterwards, a cell counting kit-8 (CCK-8, Dojindo, Minato, Japan) was used to quantify the relative metabolic activities of cells exposed to different sample extracts for 24 h. The supernatants were removed and the fibroblasts were incubated with 100 μL fresh DMEM containing 10 μL of CCK-8 reagent for 2 h at 37 °C. The optical density of samples was measured at 450 nm using a microplate reader (Tecan, Grödig, Austria).

To evaluate the direct contact cytotoxicity effect of surfaces, L929 cells were seeded at a density of 3 × 10^4^ cells/cm^2^ onto sample discs, placed in 12-well culture plates and incubated for 24 h. A live/dead staining assay was performed to evaluate the viability of cells seeded on different surfaces. After rinsing the cells with Hank’s balanced salt solution (Sigma-Aldrich, Steinheim, Germany), 1.5 mL of staining reagent, containing 1.25 μg/mL Ethidium bromide and 25 μg/mL fluorescein diacetate in HBSS (Sigma-Aldrich, Steinheim, Germany), was added. The cells were incubated for 10 min in the dark and cell viability was detected using a fluorescence microscope (Optiphot-2, Nikon, Tokyo, Japan).

### 2.4. Osteoblast Responses to Sample Surfaces

The human osteosarcoma cell line (Saos-2 osteoblast, DSMZ GmbH, Braunschweig, Germany) was cultured in McCoy’s 5A (Sigma-Aldrich, Steinheim, Germany) supplemented with 15% FBS, 1% GlutaMAX and 1% P/S under standard cell culture conditions. To determine the initial osteoblast adhesion, 3 × 10^4^ cells were seeded on the test materials for 90 min and washed with 500 µL of HBSS to remove loosely attached cells. The adherent cells were fixed with 500 µL of 3% paraformaldehyde (MERCK, Haar, Germany) in Dulbecco’s phosphate-buffered saline (DPBS, without calcium and magnesium, Gibco, Paisley, UK) for 15 min at RT. After removing the fixation buffer, the samples were stained with 500 µL crystal violet solution (2.3% crystal violet; 20% ethyl alcohol, Sigma-Aldrich) for 15 min at RT, followed by three washes with 500 µL of distilled water.

The samples were visualized using a stereomicroscope (M400, Wild Heerbrugg, Gais, Switzerland) equipped with a digital camera (EOS 500D, Canon, Tokyo, Japan). Subsequently, the crystal violet was dissolved in pure methanol (MERCK, Haar, Germany) and the absorbance was measured at 550 nm using a spectrophotometer (Tecan F50, Tecan Austria, Groedig, Austria). The mean OD values of the background controls were subtracted from the test sample values. To investigate the effects of different surfaces on osteoblast proliferation, 3 × 10^4^ Saos-2 cells were seeded on the test materials in supplemented McCoy’s 5A medium and incubated for 24 h, 48 h and 72 h under standard cell culture conditions. Relative metabolic activity was then measured using the CCK-8 assay as described before.

Additionally, alizarin Red S (ARS) staining was used to evaluate mineralized depositions as a specific marker for osteogenic maturation. Saos-2 were cultivated in osteogenic inductive medium (McCoy’s 5A medium supplemented with 100 µM L-Ascorbic acid 2-(dihydrogen phosphate) (Sigma-Aldrich A8960, Taufkirchen, Germany), 10 mM ß-Glycerophosphate disodium (Sigma-Aldrich 50020, Taufkirchen, Germany), 4 µM Dexamethasone (Sigma-Aldrich D4902, Taufkirchen, Germany)) at a density of 3 × 10^3^ cells/cm^2^, for 14 days. The medium was refreshed every 48–72 h. Following osteogenic induction, the cells were rinsed with DPBS (w/o Ca and Mg) and fixed with 3% paraformaldehyde (EMD Millipore, Germany) in DPBS for 30 min. Subsequently, the cells were washed 3 times with DPBS and stained with alizarin solution (40 mM, Sigma-Aldrich, Germany) for 30 min at 37 °C. The ARS dyes were solubilized by adding 100% ice-cold EtOH for 2 min followed by 10% acetic acid for 30 min under agitation. ARS staining intensity was quantified at a wavelength of 405 nm.

### 2.5. Hemocompatibility Testing

Heparinized whole blood (1.5 IU/mL, LEO Pharma, Neu-Isenburg, Germany) was collected from five non-medicated healthy donors. Among the exclusion criteria were patients receiving hemostasis-affecting drugs within 14 days of study entry. This procedure was approved by the Ethics Committee of the medical faculty at the University of Tübingen (Project identification code: 270/2010BO1) and all subjects gave their written informed consent for inclusion before they participated in the study. This study was conducted in accordance with the Declaration of Helsinki. An in vitro hemocompatibility test was carried out in accordance with ISO 10993-4 by evaluating blood cell counts and platelet activity. For this purpose, 11 mL of heparinized whole blood was added into a tube containing test discs and gently agitated (35 rpm) at 37 °C for 60 min. Blood cell counting was performed using a Micros 60 cell counter (ABX Hematology, Montpellier, France), both before and after incubation with the discs. The analysis of β-thromboglobulin (β-TG) was performed as previously described using ELISA (Diagnostica Stago, Asnières, France) [41]. Afterwards, samples were fixed overnight in 2% glutaraldehyde and completely dehydrated via 10 min of incubation in ascending concentrations of ethanol (40% to 100%). Dehydration was followed by critical point drying (E3100, Quorum Technologies, Laughton, UK) with liquid CO_2_. A 20 nm thick Au-Pd film (SCD 050, Baltec, Lübeck, Germany) was then sputtered onto samples, and platelet adhesion and fibrin network formation was examined using scanning electron microscopy (SEM).

### 2.6. Bacterial Interactions

The early colonizer *Streptococcus gordonii* (*S. gordonii*) strain DL1 was cultured as a stationary suspension culture overnight at 37 °C in Schaedler medium (Carl Roth GmbH, Karlsruhe, Germany). Next, the bacteria were harvested by centrifugation at 1646 g for 5 min (MegaFuge 1.0, Heraeus GmbH, Hanau, Germany) and the concentration was adjusted to OD 620 nm = 0.54 (corresponding to 1 × 10^8^ bacteria per mL) in fresh Schaedler medium. The sample disks were further incubated with 4 mL of *S. gordonii* suspension in a stirring system with continuous stop/stirring cycles (stop 10 min/stirring 2 min in each cycle) for 2 h at 37 °C. The stirring system was designed in order to simulate a dynamic flow situation, as found in the oral cavity. Following incubation, the samples were examined using either fluorescence-based live/dead staining (Bacterial Viability Kit L13152, Invitrogen, Carlsbad, CA, USA) or crystal violet staining (M400, Wild Heerbrugg, Gais, Switzerland) according to the manufacturer’s instructions. Adherent bacteria were observed using a fluorescence microscope. Finally, the crystal violet dye was solubilized and measured as described above.

### 2.7. Statistical Analysis

All data are presented as the mean and standard deviation (SD). All assays were repeated at least three times (each repeat includes four discs) to ensure reproducibility. Statistically significant differences among normally distributed data were assessed using one-way analysis of variance (ANOVA) followed by Tukey’s multiple comparisons test. Non-parametric data sets were analyzed by the Friedman test followed by Dunn’s multiple comparisons test. Statistics were analyzed using GraphPad Prism 9.4.1. Statistical significance was considered when the *p*-value was less than 0.05.

## 3. Results

### 3.1. Surface Generation and Characterization

#### 3.1.1. Surface Wettability

**Figure 1:**
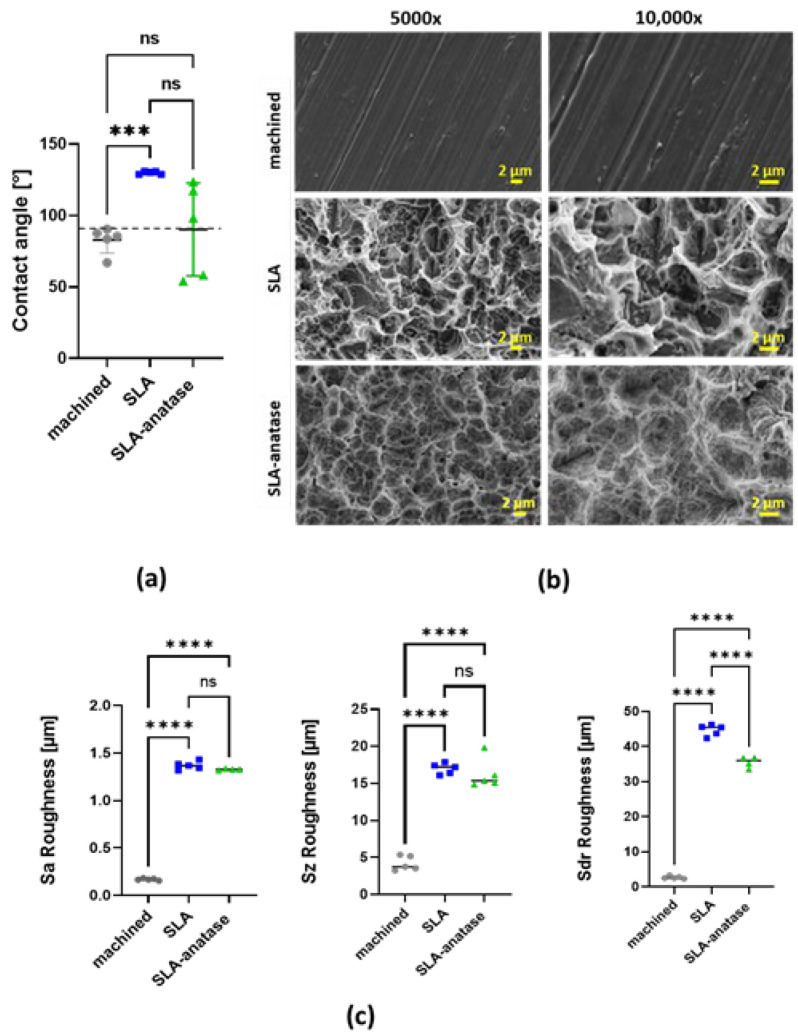
Physico-chemical characterization of surfaces (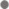: machined, 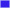: SLA, 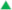: SLA-anatase). (**a**) Quantitative results of static contact angle measurements of different surfaces. The grid line represents the borderline of 90 ° between hydrophilicity and hydrophobicity, *** *p* < 0.001; (n = 5). (**b**) Surface roughness Sa of sample discs (n = 5; **** *p* < 0.0001). (**c**) Representative SEM images of the test surfaces at two different magnifications (5000× and 10,000×). SEM images demonstrate typical surface microstructures: machined, SLA and SLA-anatase surfaces. ns: not significant.

Surface wettability was evaluated by measuring static water contact angles. The mean water contact angles (n = 5) of the machined, SLA and SLA-anatase surfaces were measured as 82.7 ± 9.2°, 129.8 ± 1.0° and 90.2 ± 32.5°, respectively. The results indicate that the contact angle of the SLA disc was significantly higher than that of the machined surfaces. The surface wettability of the SLA-anatase surface did not differ when compared to the machined surface (Figure 1a).

#### 3.1.2. Morphology and Topography Analysis

Figure 1b shows representative SEM images of different surfaces. The machined surface displays characteristic streaks, which illustrate a smooth surface with cutting marks caused by polishing. SLA surfaces display a typical structure of sandblasted/acid-etched treatment, with a textured surface formed by ridges and pits with lighter and darker colors, respectively. The shaped SLA microstructures are characterized by small compartments resulting from etching on top of macrorough structures created by blasting. Correspondingly, SLA-anatase surfaces also have similar surface structures, although the pit rims appear thicker and softer.

#### 3.1.3. Surface Roughness

An optical profilometry test confirmed that SLA and SLA-anatase surfaces were rougher than machined surfaces (Figure 1c). The average roughness Sa values for the machined, SLA, and SLA-anatase surfaces were 0.16 ± 0.01 µm, 1.36 ± 0.04 µm, and 1.32 ± 0.007 µm, respectively. The average roughness values of SLA-anatase and SLA surfaces were significantly higher than those of the machined surfaces, respectively (*p* < 0.0001). Another roughness amplitude parameter, the maximum height Sz, yielded comparable results. Regarding a hybrid parameter, the developed interfacial area ratio Sdr, a statistically significant difference between SLA and SLA-anatase indicates the influence of the anatase surface modification on the additional surface area as compared to planar surfaces. This additional surface area seen on SLA was slightly reduced on SLA-anatase.

### 3.2. Cytotoxicity Testing with L929 Fibroblasts

**Figure 2:**
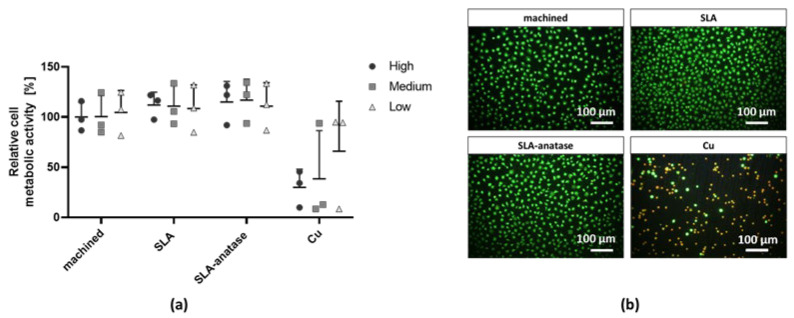
Cytotoxicity evaluation of the different surfaces after indirect or direct contact with fibroblasts in vitro. (**a**) L929 fibroblasts were cultured in sample extracts, which were added at different concentrations (high (h): 75 vol%; medium (m): 25 vol%; low (l): 7.5 vol%) to the normal cell culture media for 24 h. Relative cell metabolic activities of fibroblasts measured by CCK-8 assay are depicted. A high-machined-extract (75 vol%) medium was used as a control and set to 100%. The Cu extract served as a positive control. The data from three independent experiments are presented as bar graphs. (**b**) Representative fluorescent images after the live/dead staining of L929 fibroblasts cultivated in direct contact with the different surfaces after 24 h (100× magnified images have a scale of 100 μm).

In vitro cytotoxicity testing was performed according to ISO 10093-5:2009 after indirect or direct contact with fibroblasts. As a first step, the metabolic activity of L929 fibroblasts was investigated after the addition of extracts, which were derived by pre-incubating the medium with different test samples. Even high extract concentrations (75 vol%) did not alter cell metabolic activity in all test groups. The incubation with copper extract is the only one to show a decrease in cell metabolic activity, as expected. As compared with machined titanium surfaces, metabolic cell activity tends to be slightly increased on the SLA and SLA-anatase surfaces (Figure 2a). Furthermore, the direct cultivation of L929 fibroblasts on the sample surfaces confirms the results of the extract test (Figure 2b). The cells on the different titanium surfaces show high viability, as demonstrated by a few red-marked dead cells. A comparison of SLA-anatase and SLA surfaces to machined titanium discs also confirmed the higher cell numbers on modified surfaces (Figure 2b). Hence it can be concluded that the test materials do not induce any cytotoxic effects on fibroblasts.

### 3.3. Osteoblast Responses to Sample Surfaces

**Figure 3:**
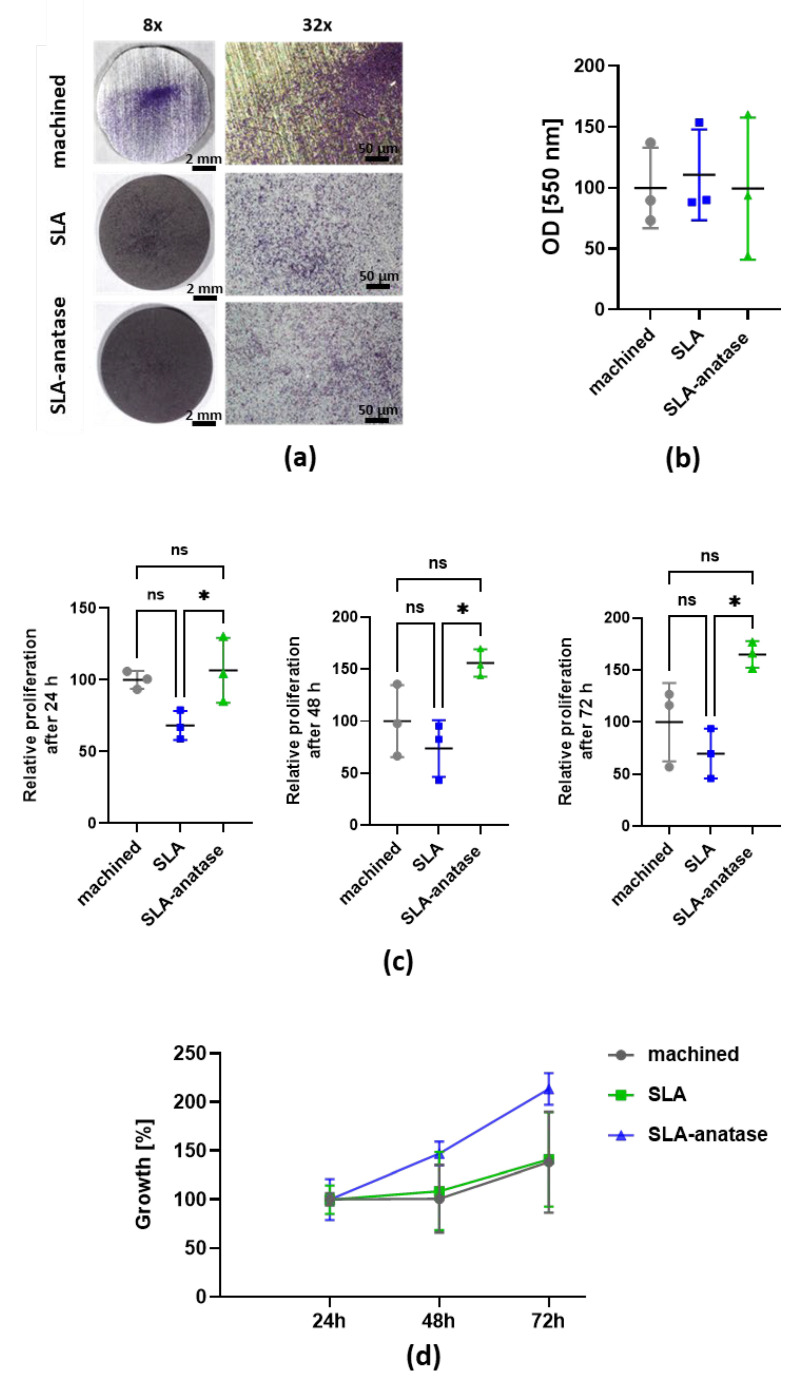
Qualitative and quantitative analysis of osteoblast adhesion and metabolic activity after incubation with the different sample surfaces. (**a**) Representative images of sample surface coverage by crystal violet stained osteoblasts after 90 min of incubation (8× and 32× magnification). (**b**) After photodocumentation, the crystal violet stain was dissolved from the cells and quantified in a spectrophotometer. The data are represented as means ± SD (n = 3). The machined group was used as a control and set to 100%. (**c**) The quantitative analysis of osteoblast metabolic activity after 24 h, 48 h and 72 h was measured by CCK-8 assay. The machined group was used as a control and set to 100% (n = 3; * *p* < 0.05). (**d**) Percentage of proliferation rate over time relative to each surface at 24 h (n = 3). ns: not significant.

Initial osteoblast adhesion to the titanium, SLA and SLA-anatase surfaces was determined using crystal violet staining after 90 min of incubation (Figure 3a). Microscopic analyses of different surfaces show no obvious differences in the number or density of cells. In addition, quantitative analysis of the crystal violet staining reveals no significant differences in cell adhesion between the surface variants after 90 min (Figure 3b).

Next, the metabolic activity of the cells was measured using the colorimetric CCK-8 assay (Figure 3c) to determine the proliferation of cells on different titanium surfaces. After 24 h, the metabolic activity of osteoblasts on the SLA-anatase surface was slightly increased when compared to the titanium control surface, and was even significantly higher when compared to the SLA surface.

This effect is even more pronounced following 48 h or 72 h incubation, and cell proliferation (on SLA-anatase surface) is significantly higher than for osteoblasts cultivated on SLA surfaces. There was no significant difference in cell metabolic activity between the titanium and SLA-anatase surfaces.

The growth of osteoblasts cultivated on SLA-anatase surfaces increased by 46.9% after 48 h and by 113.4% after 72 h (Figure 3d). In contrast, the proliferation rate of osteoblasts was reduced on the machined titanium surface (+0.6% after 48 h and +40.9% after 72 h) and the SLA surface (+8.4% after 48 h and +40.9% after 72 h).

**Figure 4:**
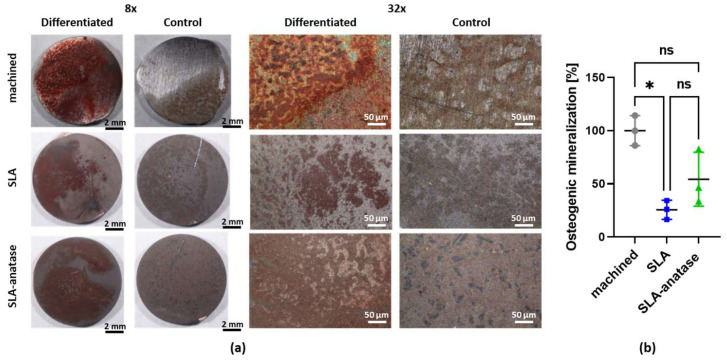
Osteogenic differentiation of osteoblasts was assessed by Alizarin red staining (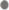: machined, 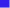: SLA, 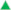: SLA-anatase). (**a**) Representative images of sample surface coverage by alizarin red stained calcium phosphate deposits after 14 days in culture under 8× and 32× magnification. (**b**) Following photodocumentation, the stain was dissolved from the cells and quantified with a spectrophotometer. The machined group was used as a control and set to 100%. The bar graph shows the mean ± SD (n = 3; * *p* < 0.05). ns: not significant.

In order to examine the ability of the different surfaces to promote osseointegration, mineralized depositions as a marker for osteoblast differentiation were analyzed after 14 days of culture. The calcium precipitates formed by the differentiated osteoblasts were stained with alizarin red, and undifferentiated cells were used as controls. Microscopically, it can be seen that most of the calcium precipitates were deposited on the machined titanium surfaces (Figure 4a). However, more calcium precipitates were formed on the SLA-anatase surfaces than on the SLA samples. These results are confirmed by the spectrophotometric quantification of the alizarin red staining by measuring the optical density of the dissolved dye (Figure 4b). The osteogenic mineralization decreased in the SLA group (*p* < 0.05) and the SLA-anatase group (not significant) compared to the titanium surface.

### 3.4. Hemocompatibility Testing

**Figure 5:**
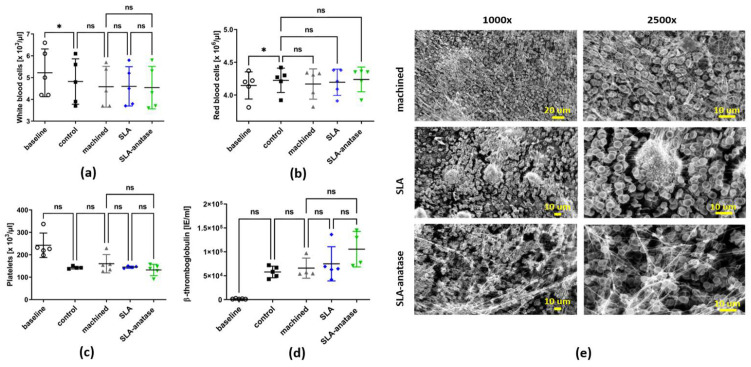
Hemocompatibility evaluation of tested surfaces in an established in vitro model using fresh human whole blood (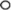: baseline, ■: control, 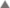: machined, 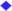: SLA, 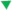: SLA-anatase). Before and after the incubation of the specimen with heparinized human whole blood for 60 min under agitation (35 rpm), white blood cells (**a**), red blood cells (**b**), platelets (**c**) and beta-thromboglobulin (**d**) were evaluated (n = 5, * *p* > 0.05). Baseline: directly after venipuncture. Control: tube only. (**e**) Representative SEM images of the three surfaces after incubation with human whole blood are depicted (magnification 1000× and 2500×). ns: not significant.

In vitro hemocompatibility tests were carried out to determine how different surface modifications interact with blood, and to identify possible unwanted interactions. Figure 5 shows the number of blood cells in different test groups following incubation in fresh blood. The results indicate that all groups maintained roughly the same numbers of leukocytes (Figure 5a) and erythrocytes (Figure 5b). These findings indicate that modified surfaces do not interact adversely with blood cells. Considering the importance of growth factors released by activated platelets in tissue regeneration, a further analysis of the number of platelets and their activation was performed. In all tested groups, free platelet numbers were lower than in the baseline, suggesting platelets adhere to titanium surfaces and well plates (Figure 5c). The platelet count was slightly lower when blood was incubated on anatase surfaces. Additionally, β-TG levels were measured to determine whether different surfaces affect platelet activation and degranulation (Figure 5d). SLA-anatase discs revealed a higher secretion of β-TG when compared to all other groups. After incubation in blood, SEM analyses were performed to assess cell deposition and protein adsorption on different modified surfaces (Figure 5e). For all tested groups, the SEM images depict uniformly arranged blood cells (mainly erythrocytes) embedded in a fine fibrin network.

### 3.5. Bacterial Interactions

**Figure 6:**
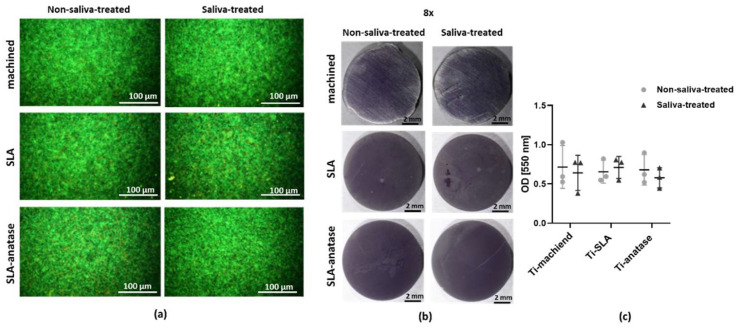
*S. gordonii* adhesion to the sample surfaces after 2 h of incubation in vitro. (**a**) Representative fluorescent microscopic images of live/dead-stained *S. gordonii* interacting with the titanium, SLA or SLA-anatase surface. The surfaces were either left untreated or pretreated with saliva. Qualitative (**b**) and quantitative (**c**) analysis after crystal violet staining of the adherent bacteria was performed (n = 3).

Various modifications of titanium surfaces were evaluated for their effects on bacterial adhesion. A live–dead staining analysis showed that there was no significant difference between the bacteria that adhered to different surfaces or to discs that had been treated with saliva or not (Figure 6a). Live bacteria were evenly distributed on all three surfaces, although dead bacteria predominated around the edges, possibly due to faster drying. Crystal violet staining of the bacteria also showed that the bacteria were spread out evenly on all three surfaces, and that samples treated with saliva and samples not treated with saliva were not significantly different (Figure 6b). These results were further confirmed by the quantitative analysis of crystal violet staining (Figure 6c).

## 4. Discussion

Titanium has proven to be an excellent implant material; it has been used in joint prostheses and dental replacements since the 1960s. Implants constructed from titanium are well tolerated by the bone and can induce osseointegration [3,5,6]. Despite high rates of implant survival and success, early implant failure occurs in 1–4% of cases mainly due to poor osseointegration [14,15,19]. The concept of surface modification involves maintaining the bulk properties of implant materials while altering the outlying surface, which interacts with surrounding tissues. Recent studies have found significant improvements in dental implant biocompatibility when using a variety of surface modifications [20,21,31]. In particular, anatase modification has been shown to improve dental implant osseointegration [34,35,42], increase osteoblast activity [31,32,33,42,43] and promote osteoblast differentiation [31,33].

The primary objective of this study was to examine the possibility of enhancing titanium osseointegration capacity by applying anatase-coated SLA compared to established pure titanium SLA implants. A thin layer of nanocrystalline anatase was coated on SLA implants by unipolar pulsed direct current (DC) sputtering. The manufactured materials were assessed with a variety of in vitro tests to evaluate their biocompatibility, physico-chemical properties, biological response and antibacterial activity.

The surface roughness of titanium dental implants is known to play a major role in both osseointegration rate and quality [29,34,35]. The wettability of the material surface is another important factor in the adhesion and activation of cells; it is therefore highly desirable to produce titanium implants with hydrophilic surfaces [8]. We examined changes in contact angles, surface roughness and surface topography to determine the physico-chemical properties of the different surfaces. SEM studies have indicated that the topography of SLA surfaces changed after TiO_2_ coating. Although the macrorough structures are still visible on SLA-anatase surfaces, the pits and rims appear thicker, which might be attributed to TiO_2_ nanostructure deposition. Further roughness analysis using optical 3D confocal microscopy revealed that the roughness of machined titanium as quantified by surface amplitude parameters significantly increased at nearly the same rate in both SLA and SLA-anatase variants. SLA and SLA-anatase could be distinguished, however, by a hybrid surface parameter that estimates the developed surface area. The hydrophobicity of pure titanium was significantly increased by applying sandblasting/acid-etching treatments. However, further anatase modification reduced the hydrophobicity of SLA surfaces. This finding indicates that the established anatase coating could improve the wetting behavior of SLA, which is desirable for cell responses. The results concerning anatase coating-related hydrophilicity agree with the findings of other research groups [44,45]. The results of our previous study have also revealed that a thin coating of the crystalline anatase modification results in a hydrophilic surface [29].

Cell–material interaction begins with protein adsorption, which is strongly influenced by the surface properties of the implanted material, such as roughness, surface charge, and wettability [46,47]. Additionally, nanoscaled biomaterials such as crystalline anatase have been shown to enhance the interaction between material and proteins that control cell adhesion and proliferation [46,47,48]. Former studies have shown that anatase-coated surfaces display good or even better surface properties when compared to other implant variants for cell interaction. Because of their high biocompatibility, anatase surfaces are associated with effective cell growth and survival [46,47,48]. Similarly, in the present study, no cytotoxic effects of different modifications on fibroblast cells were found. In addition, adhesion rates were similar on different surfaces after 24 h of incubation. These finding correspond to those of previous studies, which indicate that anatase coating has no cytotoxic effect on different cell types [46,49,50,51]. Regarding osteoblast behavior on anatase surfaces, previous studies have indicated improved cell adhesion, proliferation, and differentiation. [31,35,48]. These findings are supported by the data obtained from initial cell adhesion and metabolic activity assays in this study. In both qualitative and quantitative osteoblast adhesion after 90 min of incubation, anatase-coated titanium was comparable to the machined or SLA titanium surfaces.

The metabolic activity of osteoblasts was also evaluated in order to determine whether the tested surfaces affect osteoblast proliferation. Upon incubation for 24 h, 48 h, and 72 h, cell proliferation was surprisingly reduced on SLA surfaces. In part, this reduction can be explained by the significant increase in hydrophobicity that adversely affects the surface’s ability to interact with osteoblasts. Nevertheless, the most significant result of this study was the increase in the proliferation of cells on anatase-coated surfaces. In addition to the improved wettability of the anatase surface, roughness analysis also showed higher Sdr values on SLA-anatase, indicating a larger surface area. These factors may have contributed to the significant improvement in osteoblast proliferation on anatase-coated SLA surfaces. According to He et al., anatase-coated titanium enhances osteoblast proliferation mainly due to nanotopography, which improves surface wettability [47]. The results of our proliferation experiment are confirmed by another study, which showed that a thin layer of anatase coating over a sandblasted surface significantly increased the proliferation of osteoblast cells. It is presumed that these proliferation rates are connected to the superimposed anatase nanostructures [17].

Anatase surfaces have also been investigated for their potential for osseointegration. Based on a study by Uchida et al., titanium oxide significantly accelerates apatite formation in a simulated body fluid, with anatase showing the highest increase [28]. In another in vivo study, anatase-coated implants were found to increase bone growth with more mature bones surrounding them [34,35]. In our study, the ability of the different surfaces to promote osseointegration was evaluated in vitro based on mineralized depositions as a marker for osteoblast differentiation after 14 days of culture. Interestingly, osteoblast differentiation showed different tendencies in relation to cell adhesion and proliferation. The precipitation of calcium on machined, SLA-modified, and anatase-modified surfaces differed profoundly, with pure titanium showing the most deposition. In spite of the fact that anatase modifications tend to enhance osteoblast differentiation over SLA surfaces, pure titanium still exhibits a higher level of differentiation. A higher hydrophilicity and developed surface area may partly explain the improved osseointegration on anatase-coated surfaces over SLA. The lower calcium deposition on anatase and SLA surfaces compared with machined surfaces, however, suggests that other factors besides those influencing adhesion and proliferation affect osseointegration. Hence, to better understand these effects, further physico-chemical and biological analyses are necessary to detect other effective factors possibly involved in the osseointegration abilities of the studied surfaces.

The initial attachment of microorganisms to substrates and their subsequent retention, removal or detachment are generally affected by a variety of factors, including the chemical and physical properties of the substrate, the presence of salivary proteins and microorganisms that might foul the surface, and the nature of the interface [52]. Since osseointegration can be jeopardized by bacterial colonization-driven inflammations, antibacterial surfaces are desired on transgingival implant surfaces or in the case of recessing bone in bone-contacting implant zones. Titanium dioxide, particularly in its anatase crystalline form, has been demonstrated to have antibacterial effects both in vitro and in vivo [31,32,33,34,35,48]. Live/dead and crystal violet staining were used to assess the adhesion of an early colonizer, *S. gordonii*, to the implant surfaces. The anatase modification investigated in this study maintained bacterial adhesion levels on smooth and SLA titanium surfaces either with or without salivary macromolecular conditioning. These results are in agreement with those of similar studies that demonstrated low or no antibacterial activity in anatase coated surfaces [37,38,53,54]. According to an in vitro study, bacteria adhered less to anatase surfaces, but this change was not statistically significant [38]. Another study found no significant difference in antimicrobial behavior between uncoated and TiO_2_-coated CP-Ti implants [54]. It has also been found that bacterial adhesion to anatase-rich surfaces varies widely. The researchers proposed that a wide range of factors, including the source of bacteria (clinical isolate or lab strain), the expression levels of adhesion proteins, and saliva protein adsorption, modulated the antibacterial properties of coated surfaces. For instance, anatase exhibited low or significant antibacterial effects for laboratory strains and clinical isolates, respectively [37].

## 5. Conclusions

This study investigated the physico-chemical and biological properties of anatase-modified SLA titanium prepared by reactive pulse magnetron sputtering. Using in vitro analysis, we have demonstrated that anatase-coated implants can improve in vitro osteoblast metabolic activity compared to SLA implants without enhancing bacterial colonization. The anatase modification also partially enhanced mineralized deposition, indicating its good osseointegration properties. Further studies should be carried out in order to assess the mechanisms behind the different results of cell proliferation and differentiation, which will help in designing improved, well-functionalized biomaterials.

## Data Availability

The data presented in this study are available in the article.

## References

[1] Roberto L.L., Crespo T.S., Monteiro-Junior R.S., Martins A.M., De Paula A.M., Ferreira E.F., Haikal D.S. (2019). Sociodemographic determinants of edentulism in the elderly population: A systematic review and meta-analysis. Gerodontology.

[2] Le Guéhennec L., Soueidan A., Layrolle P., Amouriq Y. (2007). Surface treatments of titanium dental implants for rapid osseointegration. Dent. Mater..

[3] Howe M.-S., Keys W., Richards D. (2019). Long-term (10-year) dental implant survival: A systematic review and sensitivity meta-analysis. J. Dent..

[4] Kligman S., Ren Z., Chung C.-H., Perillo M.A., Chang Y.-C., Koo H., Zheng Z., Li C. (2021). The impact of dental implant surface modifications on osseointegration and biofilm formation. J. Clin. Med..

[5] Brånemark P.-I., Breine U., Adell R., Hansson B., Lindström J., Ohlsson Å. (1969). Intra-osseous anchorage of dental prostheses: I. Experimental studies. Scand. J. Plast. Reconstr. Surg..

[6] Gaviria L., Salcido J.P., Guda T., Ong J.L. (2014). Current trends in dental implants. J. Korean Assoc. Oral Maxillofac. Surg..

[7] Sykaras N., Iacopino A.M., Marker V.A., Triplett R.G., Woody R.D. (2000). Implant materials, designs, and surface topographies: Their effect on osseointegration. A literature review. Int. J. Oral Maxillofac. Implant..

[8] Rupp F., Liang L., Geis-Gerstorfer J., Scheideler L., Hüttig F. (2018). Surface characteristics of dental implants: A review. Dent. Mater..

[9] Bosshardt D.D., Chappuis V., Buser D. (2017). Osseointegration of titanium, titanium alloy and zirconia dental implants: Current knowledge and open questions. Periodontology.

[10] Hong D.G.K., Oh J.-h. (2017). Recent advances in dental implants. Maxillofac. Plast. Reconstr. Surg..

[11] McCracken M. (1999). Dental implant materials: Commercially pure titanium and titanium alloys. J. Prosthodont..

[12] Albrektsson T., Brånemark P.-I., Hansson H.-A., Lindström J. (1981). Osseointegrated titanium implants: Requirements for ensuring a long-lasting, direct bone-to-implant anchorage in man. Acta Orthop. Scand..

[13] Liu X., Chu P.K., Ding C. (2004). Surface modification of titanium, titanium alloys, and related materials for biomedical applications. Mater. Sci. Eng. R Rep..

[14] Chrcanovic B.R., Kisch J., Albrektsson T., Wennerberg A. (2018). A retrospective study on clinical and radiological outcomes of oral implants in patients followed up for a minimum of 20 years. Clin. Implant. Dent. Relat. Res..

[15] Dong H., Zhou N., Liu H., Huang H., Yang G., Chen L., Ding M., Mou Y. (2019). Satisfaction analysis of patients with single implant treatments based on a questionnaire survey. Patient Prefer. Adherence.

[16] Lu B., Zhang X., Liu B. (2021). A systematic review and meta-analysis on influencing factors of failure of oral implant restoration treatment. Ann. Palliat. Med..

[17] Liang L., Krieg P., Rupp F., Kimmerle-Müller E., Spintzyk S., Richter M., Richter G., Killinger A., Geis-Gerstorfer J., Scheideler L. (2019). Osteoblast Response to Different UVA-Activated Anatase Implant Coatings. Adv. Mater. Interfaces.

[18] Lang N.P., Salvi G.E., Huynh-Ba G., Ivanovski S., Donos N., Bosshardt D.D. (2011). Early osseointegration to hydrophilic and hydrophobic implant surfaces in humans. Clin. Oral Implant. Res..

[19] Liaw K., Delfini R.H., Abrahams J.J. (2015). Dental implant complications. Seminars in Ultrasound, CT and MRI.

[20] Silva R.C., Agrelli A., Andrade A.N., Mendes-Marques C.L., Arruda I.R., Santos L.R., Vasconcelos N.F., Machado G. (2022). Titanium dental implants: An overview of applied nanobiotechnology to improve biocompatibility and prevent infections. Materials.

[21] Barthes J., Ciftci S., Ponzio F., Knopf-Marques H., Pelyhe L., Gudima A., Kientzl I., Bognár E., Weszl M., Kzhyshkowska J. (2018). The potential impact of surface crystalline states of titanium for biomedical applications. Crit. Rev. Biotechnol..

[22] He L., Dai D., Xie L., Chen Y., Zhang C. (2021). Biological effects, applications and strategies of nanomodification of dental metal surfaces. Mater. Des..

[23] Lupi S.M., Albini B., Rodriguez y Baena A., Lanfrè G., Galinetto P. (2020). Anatase forming treatment without surface morphological alteration of dental implant. Materials.

[24] Xia W., Lindahl C., Lausmaa J., Engqvist H. (2011). Biomimetic hydroxyapatite deposition on titanium oxide surfaces for biomedical application. Adv. Biomim..

[25] Ovenstone J., Yanagisawa K. (1999). Effect of hydrothermal treatment of amorphous titania on the phase change from anatase to rutile during calcination. Chem. Mater..

[26] Wang Q.M., Zhang T.F., Kwon S.H., Kim K.H. (2011). Fabrication of TiO_2_ films on glass substrates by a pulsed dc reactive magnetron sputtering. Appl. Mech. Mater..

[27] Vahl A., Veziroglu S., Henkel B., Strunskus T., Polonskyi O., Aktas O.C., Faupel F. (2019). Pathways to tailor photocatalytic performance of TiO_2_ thin films deposited by reactive magnetron sputtering. Materials.

[28] Baptista A., Silva F., Porteiro J., Míguez J., Pinto G. (2018). Sputtering physical vapour deposition (PVD) coatings: A critical review on process improvement and market trend demands. Coatings.

[29] Rupp F., Haupt M., Klostermann H., Kim H.-S., Eichler M., Peetsch A., Scheideler L., Doering C., Oehr C., Wendel H. (2010). Multifunctional nature of UV-irradiated nanocrystalline anatase thin films for biomedical applications. Acta Biomater..

[30] Rupp F., Haupt M., Eichler M., Doering C., Klostermann H., Scheideler L., Lachmann S., Oehr C., Wendel H., Decker E. (2012). Formation and photocatalytic decomposition of a pellicle on anatase surfaces. J. Dent. Res..

[31] Hatamleh M.M., Wu X., Alnazzawi A., Watson J., Watts D. (2018). Surface characteristics and biocompatibility of cranioplasty titanium implants following different surface treatments. Dent. Mater..

[32] Rodriguez y Baena R., Rizzo S., Manzo L., Lupi S.M. (2017). Nanofeatured titanium surfaces for dental implantology: Biological effects, biocompatibility, and safety. J. Nanomater..

[33] Svetina M., Ciacchi L.C., Sbaizero O., Meriani S., De Vita A. (2001). Deposition of calcium ions on rutile (110): A first-principles investigation. Acta Mater..

[34] Uchida M., Kim H.M., Kokubo T., Fujibayashi S., Nakamura T. (2003). Structural dependence of apatite formation on titania gels in a simulated body fluid. J. Biomed. Mater. Res. Part A Off. J. Soc. Biomater. Jpn. Soc. Biomater. Aust. Soc. Biomater. Korean Soc. Biomater..

[35] Sollazzo V., Pezzetti F., Scarano A., Piattelli A., Massari L., Brunelli G., Carinci F. (2007). Anatase coating improves implant osseointegration in vivo. J. Craniofacial Surg..

[36] Scarano A., Piattelli A., Polimeni A., Di Iorio D., Carinci F. (2010). Bacterial adhesion on commercially pure titanium and anatase-coated titanium healing screws: An in vivo human study. J. Periodontol..

[37] Dorkhan M., Hall J., Uvdal P., Sandell A., Svensäter G., Davies J.R. (2014). Crystalline anatase-rich titanium can reduce adherence of oral streptococci. Biofouling.

[38] Bernardi S., Bianchi S., Botticelli G., Rastelli E., Tomei A., Palmerini M., Continenza M., Macchiarelli G. (2018). Scanning electron microscopy and microbiological approaches for the evaluation of salivary microorganisms behaviour on anatase titanium surfaces: In vitro study. Morphologie.

[39] Giordano C., Saino E., Rimondini L., Pedeferri M.P., Visai L., Cigada A., Chiesa R. (2011). Electrochemically induced anatase inhibits bacterial colonization on Titanium Grade 2 and Ti6Al4V alloy for dental and orthopedic devices. Colloids Surf. B Biointerfaces.

[40] (2012). Standardization, I. Biological Evaluation of Medical Devices—Part 12: Sample Preparation and Reference Materials.

[41] Straub A., Wendel H.P., Dietz K., Schiebold D., Peter K., Schoenwaelder S.M., Ziemer G. (2008). Selective inhibition of the platelet phosphoinositide 3-kinase p110β as promising new strategy for platelet protection during extracorporeal circulation. Thromb. Haemost..

[42] Zhang B., Li B., Gao S., Li Y., Cao R., Cheng J., Li R., Wang E., Guo Y., Zhang K. (2020). Y-doped TiO_2_ coating with superior bioactivity and antibacterial property prepared via plasma electrolytic oxidation. Mater. Des..

[43] Tang J., Wu Z., Yao X., Zhou Y., Xiong Y., Li Y., Xu J., Dargusch M.S., Yan M. (2022). From bio-inertness to osseointegration and antibacterial activity: A one-step micro-arc oxidation approach for multifunctional Ti implants fabricated by additive manufacturing. Mater. Des..

[44] Vrakatseli V., Farsari E., Mataras D. (2020). Wetting properties of transparent anatase/rutile mixed phase glancing angle magnetron sputtered nano-TiO_2_ films. Micromachines.

[45] Zhu P., Dastan D., Liu L., Wu L., Shi Z., Chu Q.-Q., Altaf F., Mohammed M.K. (2023). Surface wettability of various phases of titania thin films: Atomic-scale simulation studies. J. Mol. Graph. Model..

[46] Han W., Wang Y.D., Zheng Y. (2008). In vitro biocompatibility study of nano TiO_2_ materials. Adv. Mater. Res..

[47] Sangeetha S., Kathyayini S.R., Raj P.D., Dhivya P., Sridharan M. (2013). Biocompatibility studies on TiO2 coated Ti surface. Proceedings of the International Conference on Advanced Nanomaterials & Emerging Engineering Technologies.

[48] He J., Zhou W., Zhou X., Zhong X., Zhang X., Wan P., Zhu B., Chen W. (2008). The anatase phase of nanotopography titania plays an important role on osteoblast cell morphology and proliferation. J. Mater. Sci. Mater. Med..

[49] Cervantes B., López-Huerta F., Vega R., Hernández-Torres J., García-González L., Salceda E., Herrera-May A.L., Soto E. (2016). Cytotoxicity evaluation of anatase and rutile TiO_2_ thin films on CHO-K1 cells in vitro. Materials.

[50] Yokoi Y. (2021). Osteoblast-like Cell Proliferation, ALP Activity and Photocatalytic Activity on Sintered Anatase and Rutile Titanium Dioxide. Materials.

[51] Uboldi C., Urbán P., Gilliland D., Bajak E., Valsami-Jones E., Ponti J., Rossi F. (2016). Role of the crystalline form of titanium dioxide nanoparticles: Rutile, and not anatase, induces toxic effects in Balb/3T3 mouse fibroblasts. Toxicol. Vitr..

[52] Veerachamy S., Yarlagadda T., Manivasagam G., Yarlagadda P.K. (2014). Bacterial adherence and biofilm formation on medical implants: A review. Proc. Inst. Mech. Eng. Part H J. Eng. Med..

[53] Carballo-Vila M., Moreno-Burriel B., Chinarro E., Jurado J.R., Casañ-Pastor N., Collazos-Castro J.E. (2009). Titanium oxide as substrate for neural cell growth. J. Biomed. Mater. Res. Part A Off. J. Soc. Biomater. Jpn. Soc. Biomater. Aust. Soc. Biomater. Korean Soc. Biomater..

[54] Choi J.Y., Kim K.H., Choy K.C., Oh K.T., Kim K.N. (2007). Photocatalytic antibacterial effect of TiO_2_ film formed on Ti and TiAg exposed to Lactobacillus acidophilus. J. Biomed. Mater. Res. Part B Appl. Biomater. Off. J. Soc. Biomater. Jpn. Soc. Biomater. Aust. Soc. Biomater. Korean Soc. Biomater..

